# Immunometabolic adaptation in monocytes underpins functional changes during pregnancy

**DOI:** 10.1016/j.isci.2024.109779

**Published:** 2024-04-18

**Authors:** April Rees, Benjamin J. Jenkins, Roberto Angelini, Luke C. Davies, James G. Cronin, Nicholas Jones, Catherine A. Thornton

**Affiliations:** 1 Institute of Life Science, Swansea University Medical School, Swansea SA2 8PP, Wales, UK

**Keywords:** Reproductive medicine, Physiology, Immunology, cell biology

## Abstract

Metabolic heterogeneity is a determinant of immune cell function. The normal physiological metabolic reprogramming of pregnancy that ensures the fuel requirements of mother and baby are met, might also underpin changes in immunity that occur with pregnancy and manifest as altered responses to pathogens and changes to autoimmune disease symptoms. Using peripheral blood from pregnant women at term, we reveal that monocytes lose M2-like and gain M1-like properties accompanied by reductions in mitochondrial mass, maximal respiration, and cardiolipin content in pregnancy; glycolysis is unperturbed. We establish that muramyl dipeptide (MDP)-stimulated cytokine production relies on oxidative metabolism, then show in pregnancy reduced cytokine production in response to MDP but not LPS. Overall, mitochondrially centered metabolic capabilities of late gestation monocytes are down-regulated revealing natural plasticity in monocyte phenotype and function that could reveal targets for improving pregnancy outcomes but also yield alternative therapeutic approaches to diverse metabolic and/or immune-mediated diseases beyond pregnancy.

## Highlights

• Metabolic changes occur in pregnancy to meet the fuel requirements of mother and baby
• Immunometabolic remodeling underpins functional changes in the maternal immune system
• In human monocytes this is centered on mitochondria and OXPHOS
• It reveals natural plasticity that could be harnessed in many disease settings

## Graphical abstract

**Figure:**
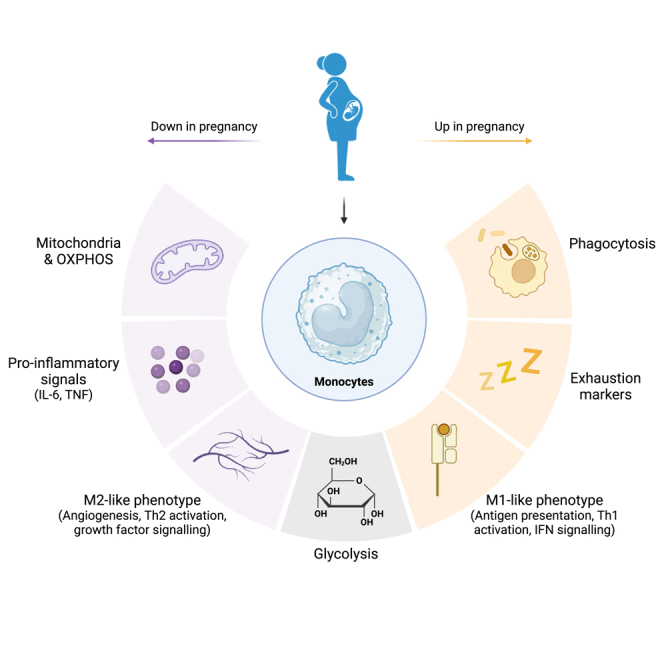

## Introduction

Pregnancy is synonymous with an altered metabolic state^1^^,^^2^ characterized by the emergence of insulin resistance and accompanying changes in carbohydrate and lipid metabolism.^1^^,^^3^ Influenced by hormones,^4^ this strategy ensures increased amino acid and glucose availability for the fetus, while meeting maternal needs by providing free fatty acids (FFAs) as an alternative energy substrate to maintain homeostasis.^5^ Accompanying these metabolic changes are a suite of dynamic immunological adaptations that occur both locally in the uterus and systemically in humoral and cellular compartments.^6^^,^^7^^,^^8^^,^^9^ Immunometabolism provides a framework to link the normal physiological changes of metabolism with pregnancy to leukocyte fate and function. As monocytes are particularly sensitive to changes in their microenvironment^10^^,^^11^^,^^12^ they provide a model cell to test this.

Monocytes also are of interest in pregnancy as likely key contributors to dynamic changes in the inflammatory balance. Alongside neutrophils, monocytes increase in the circulation as pregnancy progresses.^13^ They are also more activated during pregnancy with increased expression of activation markers, heightened intracellular ROS,^14^ and are primed to express IL-12 upon lipopolysaccharide (LPS)/IFNγ stimulation.^15^ These alterations in normal pregnancy are exacerbated in disorders such as preeclampsia,^16^ but the mechanisms driving these changes in normal and/or adverse pregnancy are poorly understood.

Given that pregnancy poses a unique risk factor for infectious disease—demonstrated by Zika virus (ZIKV),^17^ severe acute respiratory syndrome (SARS),^18^ SARS-CoV-2,^19^ and influenza,^20^ and a higher risk of developing sepsis^21^—understanding determinants of immune cell function at this time is critical. From studies in the general population, monocytes have emerged as central to the viremia that would support viral passage to the maternofoetal interface with both ZIKV and SARS-CoV-2 targeting the CD16^+^ subset of peripheral blood monocytes.^22^^,^^23^^,^^24^^,^^25^ ZIKV also targets peripheral monocytes in pregnancy leading to exacerbated M2-skewed monocytes as also seen in the general population.^26^ While not studied in pregnancy, Fcγ-mediated uptake of SARS-CoV-2 by monocytes induces pyroptosis with subsequent release of proinflammatory mediators to contribute to the pathogenesis of COVID-19.^25^ Divergent metabolic profiles of monocytes with pregnancy could therefore contribute to altered disease susceptibility of pregnant women and guide alternative approaches to limiting detrimental effects.

Many of the key features of immunometabolic adaptation by monocytes are not well studied in pregnancy but epidemiological studies and investigation of other metabolically active tissue provide some insights. Females with mitochondrial disease or dysfunction not only experience aggravation of constitutional and neurological symptoms with pregnancy but are at greater risk of adverse obstetric outcomes such as gestational diabetes mellitus (GDM), pre-eclampsia, miscarriage, and fetal congenital anomalies.^27^^,^^28^ Mitochondrial respiratory chain enzyme activity is impaired in skeletal muscle of pregnancies complicated with obesity and GDM,^29^ and high levels of anticardiolipin autoantibodies, a characteristic of antiphospholipid syndrome, occur in females with recurrent miscarriages.^30^

To date, few studies have investigated the effect of pregnancy on monocyte metabolic adaptation as a contributor to altered risk of and response to pathogens and other antigens. We hypothesized that the metabolic and hormonal changes in pregnancy contribute as driving factors for metabolic adaptation of monocytes to underpin functional changes. Here we use an immunometabolism framework to reveal the profound effects of pregnancy at term on monocyte mitochondria. Reduced mitochondrial content accompanied by reduced oxidative phosphorylation underpins a diminished response to muramyl dipeptide (MDP) but not to glycolysis dependent LPS stimulation that might alter the ability to clear pathogens and could be a target for therapeutics.

## Results

### Monocytes are phenotypically altered to adapt to the pregnant environment at term

**Figure 1:**
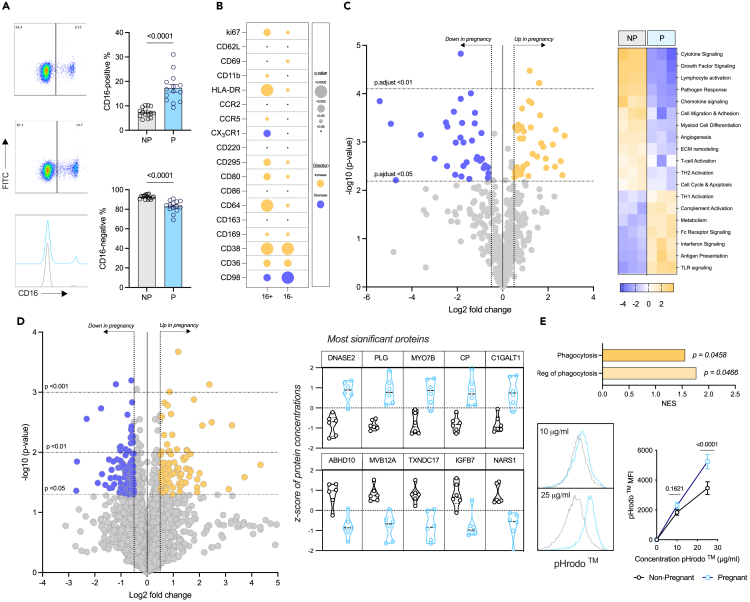
Characterizing the phenotype of monocytes in pregnancy Monocytes were isolated from the blood of non-pregnant (gray) and pregnant (blue) females for further analysis. All error bars shown are ±SEM. Flow cytometry was used to (A) identify CD16^+^ and CD16^−^subpopulations (*n* = 14/group), with a Mann-Whitney test used to determine significance (*p* < 0.05), and (B) analyze an array of expression markers on the two subsets (n = 8–12/group), with two-way ANOVA and a post-hoc Šidák’s test determining significance (*p* < 0.05). (C) Monocyte lysates were analyzed with the NanoString© Myeloid Innate Immunity panel (*n* = 3/group) to illustrate genes which were down- (purple; log fold change <0.5) or up- (gold; log fold change >0.5) regulated, and the pathways which they were associated with. (D) Proteomics (*n* = 6/group) revealed various proteins which were altered in the monocytes from pregnancy, and the top five most significant up- (log fold change >0.5) and down-regulated (log fold change <0.5) proteins are highlighted. (E) Proteins which are involved in phagocytic pathways were revealed to be up regulated with the proteomics data in the monocytes from pregnant females, and so the phagocytic capabilities of the cells were analyzed using two concentrations of pHrodo *E. coli* BioParticles : 10 and 25 μg/mL (*n* = 12). Statistical significance was determined using two-way ANOVA with a post-hoc Šidák’s test where a *p* < 0.05 was considered significant.

Monocyte activation and functional potential were examined using an extensive flow cytometry panel based on previous studies in pregnancy^14^^,^^31^ and various disease settings^32^^,^^33^ incorporating key metabolite transporters to understand immunometabolic adaptation in pregnancy. Due to the isolation process, and the low frequency of intermediates, monocyte subsets are only described here as CD16^+^ and CD16^−^as in most studies.^22^^,^^23^^,^^24^^,^^25^^,^^26^ A significant increase in CD16^+^ monocytes in pregnant females (Figure 1A) is in line with previous observations^14^^,^^34^^,^^35^ so further flow cytometry analysis was carried out on these two main subsets (Figures 1B, S1A, and S1B). Expression of the proliferation marker Ki67 on both CD16^+^ and CD16 monocytes was increased with pregnancy; unsurprising due to the overall number of monocytes increase as pregnancy progresses.^13^ Of the prototypic activation markers measured, CD62L was unchanged on both subsets, CD11b and CD69 were elevated on the CD16 subset only whereas HLA-DR was increased significantly on both CD16^+^ and CD16 monocytes. Of the chemokine receptors studied, CCR2 was unchanged but CCR5 was increased and CX3CR1 decreased on CD16^+^ monocytes with pregnancy. Despite well-recognized changes in insulin resistance with pregnancy,^3^ there was no change in insulin receptor (CD220) expression which is perhaps unsurprising given monocytes are insulin independent.^36^ Expression of the receptor for another metabolically active hormone leptin (CD295) was increased significantly on both subsets. Circulating leptin levels are increased in pregnancy^37^ with leptin resistance emerging in the second trimester.^38^^,^^39^ Leptin promotes proliferation of CD16^+^ monocytes and their phagocytic function.^40^^,^^41^

Expression of co-stimulatory CD86 was unchanged with pregnancy whereas CD80 was upregulated on both the CD16^+^ and CD16 subset, which suggests that their role as an antigen-presenting cell might be promoted in pregnancy. While expression of CD163 was unchanged with pregnancy in either subset, expression of CD64, CD169, and CD38 was increased on both subsets. CD64 can bind immunoglobulins and support phagocytosis,^42^ CD169 binds sialic acids,^43^ and CD38 is cyclic ADP ribose hydrolase.^44^ Elevation of these molecules suggests increased signal transduction, cell adhesion and phagocytosis by monocytes in pregnancy. However, studies in aging have found that CD38 levels are inversely proportional to the mitochondrial function of murine tissue cells.^45^ Expression of CD36 (roles include fatty acid transport) was increased on both subsets prompting investigation of lipid uptake and storage, but these were unchanged with pregnancy (Figure S2A). In contrast, CD98 (involved in long-chain amino acid transport) was down-regulated on both CD16^+^ and CD16 monocytes but kynurenine uptake, typically used to show the activity of CD98^46^ was increased in CD16^+^ monocytes from pregnant females at term (Figure S2B).

This phenotypic analysis not only confirms monocyte activation with pregnancy at term but that this is accompanied by likely functional and metabolic changes. Therefore, we utilized transcriptomics and proteomics to explore this further. Given the paucity of sample available to study both subsets from pregnant females separately and the shared direction of response overall, subsequent analysis was done on total CD14^+^ monocytes.

Using the NanoString© Myeloid Innate Immunity panel, we show 38 up-regulated (Table S1) and 34 down-regulated genes (Table S2) in monocytes from pregnant compared to non-pregnant females (Figure 1C). Pathway analysis demonstrated that monocytes at term appear to lose M2-like and gain M1-like properties^47^ including increases in Th1 activation, IFN signaling, and antigen presentation and decreases in angiogenesis, Th2 activation, and growth factor signaling.

To determine whether these marked changes in gene expression translate to the protein level, proteomic analysis was carried out (Figure 1D; QC plots shown in Figure S3) revealing 118 proteins significantly down-regulated and 82 significantly up-regulated. GSEA analysis was performed to determine the effect of up- (Table 1) and down-regulated (Table 2) proteins for biological processes and molecular functions. Overall, upregulation of fatty acid metabolism and phagocytosis alongside a pro-thrombotic signature, that likely reflects the hypercoagulation state of pregnancy,^48^ emerges in monocytes with pregnancy. Processes that were down-regulated include regulation of translation and functional GTPase activity suggesting poor translational capabilities of the cells and explains discrepancies between the mRNA data generated from the NanoString© and the protein data.

**Table 1:** Significantly up-regulated biological processes and molecular functions from the GSEA analysis of the proteomics data

	ID	Description	NES	p.adjust
**Biological processes**	GO:0042060	wound healing	2.275	1.886E-07
	GO:0007596	blood coagulation	2.390	1.9012E-07
	GO:0007599	hemostasis	2.391	1.9012E-07
	GO:0050817	coagulation	2.394	1.9012E-07
	GO:0009611	response to wounding	2.052	4.3347E-07
	GO:0006959	humoral immune response	2.288	2.2782E-05
	GO:0042742	defense response to bacterium	2.133	2.7112E-05
	GO:0019730	antimicrobial humoral response	2.373	0.0002
	GO:0050878	regulation of body fluid levels	1.885	0.0002
	GO:0009617	response to bacterium	1.699	0.0003
	GO:1903034	regulation of response to wounding	2.140	0.0009
	GO:0061041	regulation of wound healing	2.223	0.0014
	GO:0030168	platelet activation	2.057	0.0020
	GO:0050818	regulation of coagulation	2.184	0.0037
	GO:0006631	fatty acid metabolic process	1.728	0.0038
	GO:0061045	negative regulation of wound healing	2.092	0.0061
	GO:0002250	adaptive immune response	1.635	0.0071
	GO:0030193	regulation of blood coagulation	2.156	0.0071
	GO:1900046	regulation of hemostasis	2.156	0.0071
	GO:0010951	negative regulation of endopeptidase activity	1.768	0.0076
	GO:1903035	negative regulation of response to wounding	2.050	0.0076
	GO:0010466	negative regulation of peptidase activity	1.748	0.0080
	GO:0070527	platelet aggregation	1.926	0.0126
	GO:0051346	negative regulation of hydrolase activity	1.604	0.0131
	GO:0003018	vascular process in circulatory system	1.821	0.0143
	GO:0031589	cell-substrate adhesion	1.666	0.0153
	GO:1903409	reactive oxygen species biosynthetic process	1.953	0.0216
	GO:0002526	acute inflammatory response	2.014	0.0241
	GO:0098609	cell-cell adhesion	1.408	0.0246
	GO:0035150	regulation of tube size	1.936	0.0246
	GO:0035296	regulation of tube diameter	1.936	0.0246
	GO:0097746	blood vessel diameter maintenance	1.936	0.0246
	GO:0022900	electron transport chain	1.636	0.0253
	GO:0002252	immune effector process	1.387	0.0258
	GO:0048525	negative regulation of viral process	1.763	0.0258
	GO:0034109	homotypic cell-cell adhesion	1.849	0.0258
	GO:0006911	phagocytosis, engulfment	1.862	0.0258
	GO:0006956	complement activation	1.959	0.0258
	GO:0031638	zymogen activation	1.877	0.0299
	GO:0050865	regulation of cell activation	1.401	0.0342
	GO:0099024	plasma membrane invagination	1.765	0.0345
	GO:0051702	biological process involved in interaction with symbiont	1.826	0.0444
	GO:0006909	phagocytosis	1.556	0.0458
	GO:0007160	cell-matrix adhesion	1.608	0.0466
	GO:0050764	regulation of phagocytosis	1.766	0.0466
	GO:0002443	leukocyte mediated immunity	1.476	0.0477
**Molecular functions**	GO:0003823	antigen binding	2.422	0.0003
	GO:0003735	structural constituent of ribosome	1.875	0.0004
	GO:0004866	endopeptidase inhibitor activity	1.910	0.0040
	GO:0030414	peptidase inhibitor activity	1.910	0.0040
	GO:0061135	endopeptidase regulator activity	1.831	0.0060
	GO:0009055	electron transfer activity	1.698	0.0093
	GO:0008201	heparin binding	1.931	0.0093
	GO:0061134	peptidase regulator activity	1.679	0.0128
	GO:0016853	isomerase activity	1.653	0.0129
	GO:0016651	oxidoreductase activity, acting on NAD(P)H	1.747	0.0189
	GO:0050660	flavin adenine dinucleotide binding	1.794	0.0189
	GO:0051087	chaperone binding	1.672	0.0214
	GO:0005178	integrin binding	1.779	0.0214
	GO:0002020	protease binding	1.657	0.0220
	GO:0005539	glycosaminoglycan binding	1.707	0.0220
	GO:0030246	carbohydrate binding	1.504	0.0241
	GO:0003955	NAD(P)H dehydrogenase (quinone) activity	1.736	0.0241
	GO:1901681	sulfur compound binding	1.575	0.0379
	GO:0004867	serine-type endopeptidase inhibitor activity	1.674	0.0391

**Table 2:** Significantly down-regulated biological processes and molecular functions from the GSEA analysis of the proteomics data

	ID	Description	NES	p.adjust
**Biological processes**	GO:1903311	regulation of mRNA metabolic process	−1.873	2.23E-05
	GO:1903313	positive regulation of mRNA metabolic process	−1.982	0.0003
	GO:0050684	regulation of mRNA processing	−1.894	0.0009
	GO:0043484	regulation of RNA splicing	−1.840	0.0017
	GO:0000380	alternative mRNA splicing, via spliceosome	−1.912	0.0038
	GO:0006403	RNA localization	−1.720	0.0038
	GO:0010638	positive regulation of organelle organization	−1.608	0.0049
	GO:0048024	regulation of mRNA splicing, via spliceosome	−1.832	0.0076
	GO:0051028	mRNA transport	−1.782	0.0076
	GO:0006417	regulation of translation	−1.560	0.0083
	GO:1902117	positive regulation of organelle assembly	−1.852	0.0107
	GO:1902115	regulation of organelle assembly	−1.808	0.0107
	GO:0000381	regulation of alternative mRNA splicing, via spliceosome	−1.816	0.0143
	GO:0043487	regulation of RNA stability	−1.712	0.0159
	GO:0006402	mRNA catabolic process	−1.647	0.0159
	GO:0043488	regulation of mRNA stability	−1.730	0.0203
	GO:0120032	regulation of plasma membrane bounded cell projection assembly	−1.720	0.0246
	GO:0032388	positive regulation of intracellular transport	−1.676	0.0246
	GO:0050657	nucleic acid transport	−1.624	0.0246
	GO:0050658	RNA transport	−1.624	0.0246
	GO:0043009	chordate embryonic development	−1.587	0.0246
	GO:0016050	vesicle organization	−1.574	0.0246
	GO:0009792	embryo development ending in birth or egg hatching	−1.565	0.0246
	GO:0061157	mRNA destabilization	−1.743	0.0247
	GO:0061014	positive regulation of mRNA catabolic process	−1.750	0.0258
	GO:0061013	regulation of mRNA catabolic process	−1.694	0.0258
	GO:0044089	positive regulation of cellular component biogenesis	−1.529	0.0258
	GO:0000377	RNA splicing, via transesterification reactions with bulged adenosine as nucleophile	−1.513	0.0258
	GO:0000398	mRNA splicing, via spliceosome	−1.513	0.0258
	GO:0000375	RNA splicing, via transesterification reactions	−1.498	0.0258
	GO:0006401	RNA catabolic process	−1.522	0.0287
	GO:0050779	RNA destabilization	−1.730	0.0299
	GO:0051236	establishment of RNA localization	−1.610	0.0342
	GO:0017148	negative regulation of translation	−1.603	0.0400
	GO:0120031	plasma membrane bounded cell projection assembly	−1.547	0.0418
	GO:0016573	histone acetylation	−1.659	0.0452
	GO:0030031	cell projection assembly	−1.572	0.0456
	GO:0018105	peptidyl-serine phosphorylation	−1.550	0.0458
**Molecular functions**	GO:0045296	cadherin binding	−1.696	0.0007
	GO:0008022	protein C-terminus binding	−1.846	0.0015
	GO:0005096	GTPase activator activity	−1.668	0.0024
	GO:0030695	GTPase regulator activity	−1.625	0.0024
	GO:0060589	nucleoside-triphosphatase regulator activity	−1.625	0.0024
	GO:0035091	phosphatidylinositol binding	−1.699	0.0056
	GO:0003729	mRNA binding	−1.568	0.0124
	GO:0005085	guanyl-nucleotide exchange factor activity	−1.632	0.0129
	GO:1901981	phosphatidylinositol phosphate binding	−1.674	0.0157
	GO:0051020	GTPase binding	−1.535	0.0189
	GO:0003730	mRNA 3′-UTR binding	−1.715	0.0220
	GO:0016779	nucleotidyltransferase activity	−1.669	0.0233
	GO:0140098	catalytic activity, acting on RNA	−1.499	0.0233
	GO:0045309	protein phosphorylated amino acid binding	−1.677	0.0314
	GO:0140142	nucleocytoplasmic carrier activity	−1.664	0.0348
	GO:0106310	protein serine kinase activity	−1.485	0.0379
	GO:0106306	protein serine phosphatase activity	−1.637	0.0383
	GO:0106307	protein threonine phosphatase activity	−1.637	0.0383
	GO:0031267	small GTPase binding	−1.506	0.0460
	GO:0008135	translation factor activity, RNA binding	−1.566	0.0499

Given the upregulation of CD16, CD36, and CD64 with the proteomics data which shows an upregulation of proteins involved in phagocytosis and the regulation of phagocytosis in monocytes of pregnant females, the phagocytic ability of monocytes was evaluated and showed increased phagocytosis of pHrodo red *E. coli* BioParticles (Figure 1E). Taken together these data demonstrate that monocytes are phenotypically and functionally altered by 37+ weeks of gestation.

### Monocytes in pregnancy at term are significantly altered metabolically

**Figure 2:**
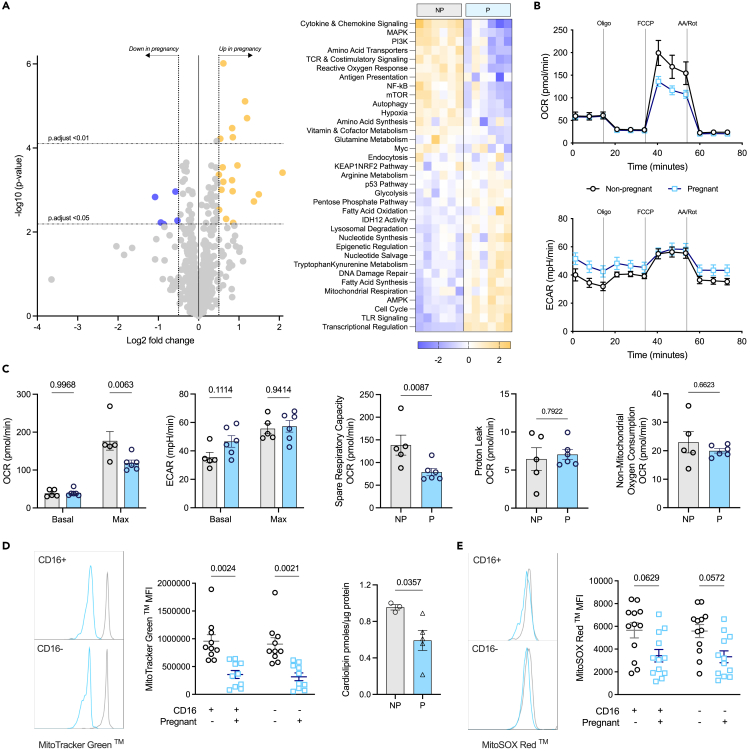
The metabolism of monocytes in pregnancy All error bars shown are ±SEM. (A) Isolated monocyte lysates were analyzed using the NanoString© Metabolism Pathways panel (*n* = 6/group) to reveal genes relating to metabolism which were down- (purple; log fold change <0.5) and up- (gold; log fold change >0.5) regulated, and the pathways the genes contribute to. (B) Monocytes from non-pregnant (gray; *n* = 5) and pregnant (blue; *n* = 6) females were run on the Seahorse Bioscience XFe96 Extracellular Flux Analyzer in the presence of injections used to target components of the electron transport chain: oligomycin (oligo), FCCP, and antimycin A (AA), and rotenone (R). (C) Oxygen consumption rate (OCR) and extracellular acidification rate (ECAR) were measured to determine basal and maximal OXPHOS and glycolysis respectively, along with other related metabolic measures such as spare respiratory capacity, proton leak, and non-mitochondrial oxygen consumption. (D) Mitochondrial content was determined using MitoTracker Green (*n* = 10) via flow cytometry and cardiolipin (n = 3–5/group) content via MALDI-ToF MS; statistical analysis was with either a two-way ANOVA with a post-hoc Šidák’s test, or Mann-Whitney test respectively, where *p* < 0.05 was considered significant. (E) The use of MitoSOX Red (*n* = 12/group) with flow cytometry determined the mitochondrial superoxide production of the monocytes; statistical analysis was performed as with MitoTracker staining.

With changes in metabolic transporter expression (Figure 1B), significantly increased expression of multiple metabolism-related genes revealed using the NanoString© Myeloid Innate Immunity panel (Figure 1C), and some of the top five proteins significantly down-regulated being involved with mitochondrial ROS clearance and the metabolism of macromolecules, the metabolism of monocytes was considered more closely. Using a NanoString © nCounter Metabolism Pathways panel (Figure 2A), we discovered 5 genes that were significantly down-regulated in monocytes from pregnancy, and 19 which were up-regulated (Table S3). Analysis of the pathway scores highlighted significant down-regulation of genes relating (but not limited) to *mTOR* and *Myc*, and overexpression of genes involved in *AMPK* and mitochondrial respiration.

Considering this, bioenergetic analysis was carried out on the monocytes using the MitoStress test on the Seahorse Extracellular Flux Analyzer (Figures 2B and 2C). Maximal oxidative phosphorylation (OXPHOS) was found to be decreased significantly in monocytes from pregnant females, along with the spare respiratory capacity. No changes in their glycolytic capabilities were observed. This prompted analysis of the mitochondrial mass of the cells using MitoTracker Green and revealed a significant decrease in mitochondrial content with pregnancy that was further confirmed with analysis of cardiolipin which is the signature lipid of mitochondrial membranes (Figure 2D). While the production of mitochondrial superoxide (measured by MitoSOX Red) was decreased with pregnancy (Figure 2E) this did not reach significance. Overall, we show that monocytes from term pregnancy have reduced mitochondrial content leading to decreased OXPHOS.

### MDP-stimulated monocytes outside of pregnancy rely on oxidative phosphorylation

**Figure 3:**
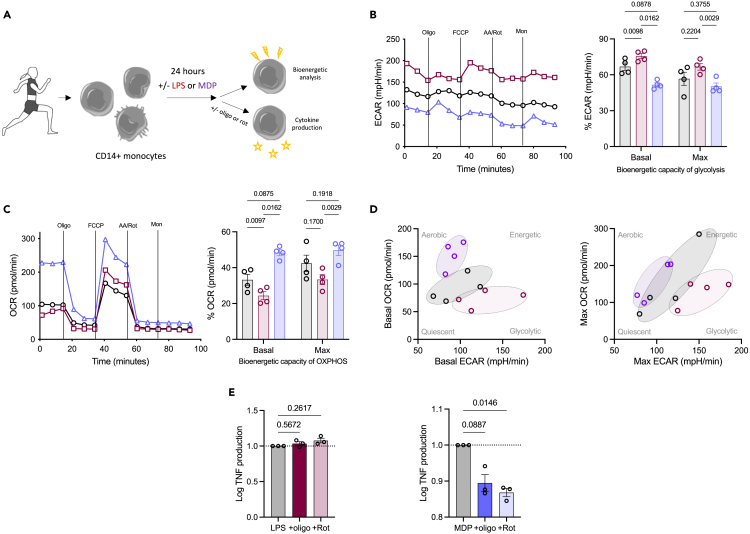
Identifying a PAMP which is OXPHOS-dependent All error bars shown are ±SEM. (A) Monocytes were isolated from the blood of healthy non-pregnant females and subjected to LPS (10 ng/mL; red) or MDP (1 μg/mL; purple) treatment or left untreated (black) for 24 h before bioenergetic analysis; alternatively mitochondrial inhibitors were added with the initial stimuli for the measurement of cytokine production. Bioenergetic analysis (*n* = 4/group) measures the (B) oxygen consumption rate (OCR) and (C) extracellular acidification rate (ECAR) to reveal the basal and maximal bioenergetic capacity of OXPHOS and glycolysis, respectively. Statistical analysis was with a two-way ANOVA with a post-hoc Šidák’s test, where *p* < 0.05 was considered significant. (D) Energy distribution maps for basal and maximal respiration illustrate the clustering of the different conditions based on their OCR and ECAR. (E) Monocytes were also cultured with LPS or MDP for 24 h in the presence of oligomycin (oligo) or rotenone (rot) and TNF production was analyzed using ELISA (*n* = 3). Statistical analysis was performed using a one-way ANOVA with a post-hoc Dunnett’s test, where *p* < 0.05 was considered significant.

To determine the functional consequences of reduced OXPHOS, we then sought a functional readout dependent on this metabolic pathway for further analysis. While LPS is a common stimulus used to induce a response from monocytes, it is known to stimulate a switch to glycolysis in monocytes.^49^ It has been suggested that MDP, a peptidoglycan constituent of both gram positive and negative bacteria and recognized intracellularly by NOD2, targets mitochondrial respiration in murine macrophages.^50^ Therefore, we explored if MDP might be an OXPHOS-dependent PAMP (Figure 3A).

Monocytes from non-pregnant donors were stimulated with either LPS or MDP or left unstimulated for 24 h prior to analysis of their bioenergetic capabilities. As expected,^49^^,^^51^ LPS-stimulated monocytes show marked upregulation of glycolysis (Figure 3B) whereas original data show MDP-stimulated monocytes rely heavily on OXPHOS (Figure 3C). Using energy distribution maps to illustrate this more clearly, it is evident that for both basal and maximal respiration, LPS-stimulated monocytes shift to be more glycolytic, whereas MDP-stimulated monocytes shift to be more aerobic, with the unstimulated monocytes sitting in between (Figure 3D).

To confirm a reliance of MDP-stimulated monocytes on OXPHOS for their functional outputs, LPS- and MDP-stimulated monocytes were cultured for 24 h with and without the mitochondrial inhibitors oligomycin (inhibitor of ATP synthase) or rotenone (inhibitor of complex I; Figure 3E). Inhibiting mitochondrial respiration had no effect on LPS-stimulated TNF production but both oligomycin and rotenone inhibited the secretion of TNF from MDP-stimulated monocytes. These data demonstrated monocytes have a functional reliance on OXPHOS when stimulated with MDP.

### Cytokine production is impaired in MDP-stimulated monocytes from pregnant females at term

**Figure 4:**
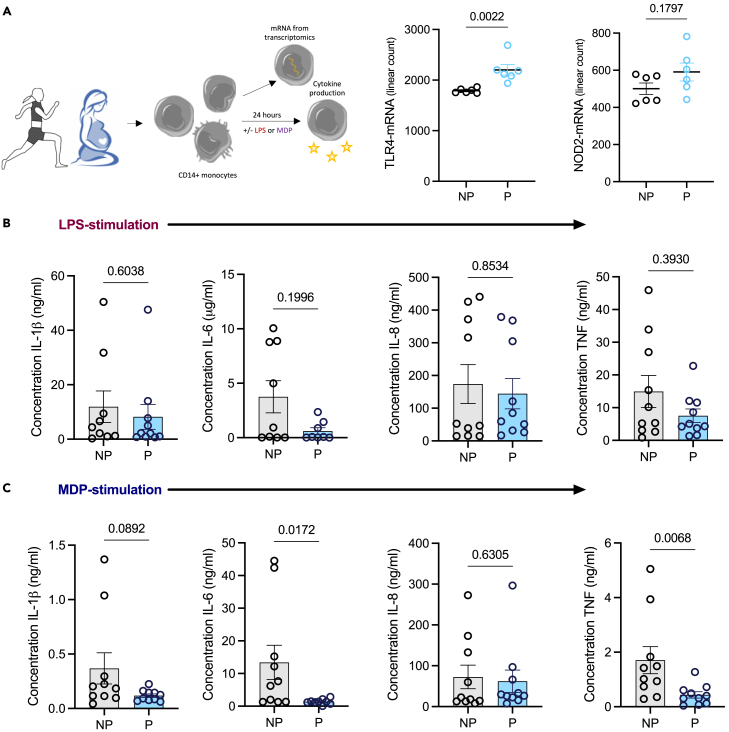
Cytokine production is impaired in MDP-stimulated monocytes from pregnant females All error bars shown are ±SEM. (A) mRNA counts from the NanoString ^(C)^ Metabolism Pathways panel (*n* = 6) were extracted for TLR4 and NOD2. ELISAs (n = 8–10) were performed as in the materials and methods analyzing the concentration of IL-1β, IL-6, IL-8, and TNF for (B) LPS- and (C) MDP-stimulated monocytes. Statistical analysis was performed using a Mann-Whitney test where *p* < 0.05 was considered significant.

We next considered the functional consequences of diminished mitochondrial content for inflammatory cytokine output from monocytes derived from pregnant versus non-pregnant females. Firstly, TLR4 and NOD2 gene expression (pattern recognition receptors [PRRs] for LPS and MDP respectively) was extrapolated from the NanoString© data to ensure that any changes observed were metabolically linked and not due to reduced expression of the PRR; accordingly, TLR4 was significantly upregulated whereas NOD2 was unchanged in monocytes from pregnant females (Figure 4A). We observed no significant difference in IL-1β, IL-6, IL-8, and TNF levels from LPS-stimulated monocytes (Figure 4B) from pregnant in comparison to non-pregnant females. However, MDP-stimulated monocytes demonstrated a significant reduction in IL-6 and TNF (Figure 4C) from the pregnant females. This illustrates that metabolic adaptation of monocytes cantered on mitochondrial content has significant functional consequences with reduced capability to mount an inflammatory response.

## Discussion

Immunometabolism might offer a global mechanism underpinning immune plasticity in pregnancy linking well described changes in substrate availability with changes to immune cell function in both healthy and adverse pregnancy. Monocytes from pregnant females at term are clearly altered phenotypically and metabolically evidencing adaptation to the late pregnancy environment. This is apparent at the transcript, protein, and functional level. The most striking discovery is the reduced mitochondrial content that is accompanied by a stark difference in the OXPHOS of the monocytes which leads to diminished function.

Initial phenotypic analysis confirmed an increase in CD16^+^ monocytes with pregnancy and an activated phenotype.^14^^,^^34^ The detailed flow cytometric analysis of CD16^+^ and CD16 subsets in contrast to all previous studies on total monocytes only, provided unparalleled insight into the effects of pregnancy on these two key monocyte subsets, extending the activated phenotype to include elevated HLA-DR, CD11b, and CD69 and providing evidence of likely functional alterations linked to increased CD38, CD64, and CD169. From a metabolic perspective, the leptin (CD295) but not insulin (CD220) receptor was elevated with pregnancy. Pregnancy is a leptin-rich environment^37^ and leptin has been shown to promote the expansion of CD16^+^ monocytes and their phagocytic activity,^41^ as well as increasing CD36 expression, changes which are observed here.^52^ Along with the changes in CD36, CD98 expression was reduced on the monocytes in pregnancy, suggesting metabolic changes to the cells.

A combination of transcriptomics and proteomics provided deeper insight. Transcriptomics highlighted an overall deviation from M2-like properties such as angiogenesis and OXPHOS, to M1-like properties such as TLR signaling and phagocytosis. This is in parallel to the changes observed with CD80, one of the T cell co-stimulatory counter receptors which was found to be significantly elevated on both subsets of monocytes in pregnancy, suggesting a heightened inclination to induce T cell activation at this stage of pregnancy. Pathway scores showed that genes involved in the mTOR pathway are decreased significantly concurrent with an increase in those involved in the AMPK pathway meaning a likely sum effect of reduced oxygen consumption and ATP synthetic capacity.^53^ This was accompanied by an increase in the genes involved in mitochondrial respiration. Bioenergetics analysis revealed that monocytes from pregnant females have diminished maximal OXPHOS in keeping with the changes to the mTOR and AMPK pathways. Reduced mitochondrial content of monocytes from pregnant females also provides an explanation for these changes. Complexes III and IV require cardiolipin molecules to maintain their structure,^54^ and these were decreased in the monocytes in pregnant women. ROS are a by-product of OXPHOS, and these were found to be reduced by pathway score for the reactive oxygen response and by flow cytometry for mitochondrial ROS supporting a profound effect of late pregnancy on mitochondrial content and OXPHOS. With LPS being shown by others^51^ and here to be glycolysis dependent, we establish the oxidative dependence of MDP and have then used this to show the effects of reduced mitochondrial content in pregnancy manifests as perturbed cytokine output on MDP but not LPS stimulation.

Reduced mitochondrial content and elevated CD38, as described here, are considered hallmarks of monocyte exhaustion.^55^ Elevated STAT1 is also a hallmark of exhaustion.^55^ Interrogation of both the transcriptomic and proteomic datasets revealed elevated monocyte STAT1 mRNA and protein with pregnancy (Figure S4A). As prolonged exposure to LPS can lead to monocyte exhaustion and CD38 expression is advanced by LPS, which is commonly found in peripheral blood,^56^^,^^57^ the possibility that LPS itself was a driver of this emergent exhaustion phenotype was considered. LPS binding-protein (LBP) provides a surrogate of LPS levels^58^^,^^59^ and is significantly elevated in the circulation of pregnant females (Figure S4B). This likely reflects increased gut permeability with pregnancy.^60^^,^^61^ Here, we propose that by the end of full-term pregnancy monocytes have developed an exhausted phenotype which impacts their functional responses and explains some of the increased risk to infectious diseases.^19^^,^^62^ To fully understand this, we now need prospective analysis from the same women across the course of pregnancy and consideration of a single time point is a limitation of this study.

Possible maladaptation of the physiologically normal, mitochondrially centered change in metabolism and the accompanying exhaustion phenotype that we have revealed here would now be interesting to explore in settings such as gestational diabetes and obesity. Evidence of metabolic adaptation and dysregulation of this that can be made using easily accessible peripheral blood rather than skeletal muscle, adipose tissue, or liver, makes such studies in humans now more feasible.^27^^,^^28^^,^^29^ We have already shown that at 28 weeks of gestation, obesity is associated with decreased mitochondrial content and activation marker expression in monocytes as well as mild intrauterine growth restriction at term.^63^ Metabolic dysregulation such as type 1 and type 2 diabetes mellitus (DM) predispose pregnant women to various fetal (e.g., macrosomia, congenital malformations, injury, and preterm delivery) and maternal (e.g., retinopathy, neuropathy, nephropathy, and hypertension) risks. Little is known about the effect of these, or GDM, on the monocytes during pregnancy but PBMCs in non-pregnant T2DM have illustrated reduced mitochondrial mass, hyperpolarised mitochondria, and increased mitochondrial superoxide production.^64^ Hyperglycaemia has also been shown to induce excessive superoxide production by the ETC, causing oxidative stress in aortic endothelial cells, and inducing a decrease in GAPDH activity.^65^ This suggests that the monocytes could have a term-like state as we describe here at the beginning of pregnancy, leading to poor fetal and placental development, and poor outcomes. Further activation of monocytes is synonymous with preeclampsia, as well as elevated non-classical monocytes^16^; it has been suggested that these highly active monocytes invade the decidua to develop into M1-like macrophages, further promoting a pro-inflammatory environment, stressing the placenta and inhibiting spiral artery remodeling.^16^ It is therefore key to understand the healthy adaptations of the monocytes and macrophages in pregnancy, before considering adverse outcomes, and how they could potentially be targeted therapeutically.

### Conclusion

Pregnancy programmes late gestation monocytes to influence their environment by dampening their metabolic abilities and generating an exhausted phenotype. This is evidenced at the transcript, protein, and functional level. The most striking discovery is the reduced mitochondrial content of the monocytes, which translates to a reduction in their OXPHOS capabilities and results in diminished function, evidenced when stimulated with MDP but not LPS. The functional ramifications of this are well documented in an extensive literature of altered disease resistance in pregnant women.

### Limitations of the study

Ethically, we were only able to obtain approximately 35 mL of peripheral blood from the pregnant participants, so were limited in what could be done with any one donor by the number of cells any methodology requires so not all experiments were possible on the same individual. Wherever possible, we have performed multiple experiments on the cells from the same individual. To fully establish the changes to monocytes over the course of pregnancy, the ideal approach would be longitudinal studies. However, this is very time consuming as we would ideally study the same women at different times of pregnancy, including a prior-to-conception sample, and then only include data from women who had normal pregnancies—any adverse outcomes would of course provide anecdotal insight into the contribution of metabolic maladaptation of these. Here, we have chosen to focus on late gestation when any pregnancy associated phenotype is likely to be strongest to first ascertain if such a study would be warranted.

## STAR★Methods

### Key resources table

**Table:** 

REAGENT or RESOURCE	SOURCE	IDENTIFIER
**Antibodies**		

CD11b human antibody (clone CBRM1/5)	BioLegend	Cat#301406; RRID: AB_314170
CD14 human antibody (clone 63D3)	BioLegend	Cat#367122; RRID: AB_2687384
CD16 human antibody (clone VEP13)	Miltenyi	Cat#130-099-080; RRID: AB_2661279
CD16 human antibody (clone REA423)	Miltenyi	Cat#130-113-392; RRID: AB_2726150
CD36 human antibody (clone 5-271)	BioLegend	Cat#336206; RRID: AB_2072513
CD38 human antibody (clone HB-7)	BioLegend	Cat#356604; RRID: AB_2561900
CD62L human antibody (clone DREG-56)	BioLegend	Cat#304806; RRID: AB_314466
CD64 human antibody (clone 10·1·1)	Miltenyi	Cat#130-124-234; RRID: AB_2857679
CD69 human antibody (clone FN50)	BioLegend	Cat#310906; RRID: AB_314841
CD80 human antibody (clone 2D10)	Miltenyi	Cat#130-117-683; RRID: AB_2733839
CD86 human antibody (clone FM95)	Miltenyi	Cat#130-113-572; RRID: AB_2733843
CD98 human antibody (clone UM7F8)	BD Horizon	Cat#556076; RRID: AB_396343
CD163 human antibody (clone GHI/61.1)	Miltenyi	Cat#130-123-249; RRID: AB_2819455
CD169 human antibody (clone 7-239)	Miltenyi	Cat#130-098-654; RRID: AB_2655545
CD192/CCR2 human antibody (clone REA624)	Miltenyi	Cat#130-109-595; RRID: AB_2655867
CD195/CCR5 human antibody (clone REA245)	Miltenyi	Cat#130-117-356; RRID: AB_2733783
CD220 human antibody (clone REA260)	Miltenyi	Cat#130-126-493; RRID: AB_2889508
CD295/LEPR human antibody (clone REA361)	Miltenyi	Cat#130-125-203; RRID: AB_2889537
CX3CR1 human antibody (clone 2A9-1)	Miltenyi	Cat#130-096-432; RRID: AB_10828443
HLA-DR human antibody (clone L243)	BioLegend	Cat#307636; RRID: AB_2561831

**Biological samples**		

Blood obtained from healthy adult donors (females aged 18-40)	N/A	REC 13/WA/0190
Blood obtained from pregnant patients at 37+ Weeks prior to C-section	N/A	REC 11/WA/0040

**Chemicals, peptides, and recombinant proteins**		

2-Amino-2-norbornanecarboxylic acid (BCH)	Merck	A7902
2-Propanol	Fisher Scientific	11325327
9-aminoacridine	Merck	92817
Acetonitrile	Fisher Scientific	10794741
Acetonitrile ≥99.9%, HiPerSolv CHROMANORM® for LC-MS, suitable for UPLC/UHPLC instruments	VWR	83640.290
Antimycin A	Merck	A8674
Avanti Polar Lipids 14:0 Cardiolipin	Merck	710332P
Buffer RLT	Qiagen	79216
Carbonyl cyanide-p-trifluoromethoxyphenylhydrazone (FCCP)	Merck	C2920
Cell-Tak™ Cell and Tissue Adhesive	Corning	354240
DTT (dithiothreitol)	Thermo Scientific	R0861
Formic Acid, 99.0+%, Optima™ LC/MS Grade, Fisher Chemical™	Fisher Scientific	10596814
Iodoacetamide	Merck	I6125
L-Kynurenine	Tocris	4393/50
L-Lysine	Merck	L5501
LPS-EB Ultrapure	Invivogen	tlrl-3pelps
Lymphoprep™	StemCell Technologies	7861
Methanol ≥99.9% (by GC), HiPerSolv CHROMANORM® for LC-MS, suitable for UPLC/UHPLC instruments	VWR	83638.29
Methanol ≥99.9% (by GC), HiPerSolv CHROMANORM® for LC-MS, suitable for UPLC/UHPLC instruments	VWR	83638.29
Monensin	Merck	M5273
Muramyldipeptide (L-D isoform, active)	Invivogen	tlrl-mdp
Oligomycin	Merck	75351
Orthophosphoric acid ≥85%, AnalaR NORMAPUR® ACS, Reag. Ph. Eur. analytical reagent	VWR	20624.262
Paraformaldehyde (PFA)	Merck	8.18715
Rotenone	Merck	R8875
Sodium dodecyl sulfate (SDS)	Merck	L4509
Triethylammonium bicarbonate buffer	Merck	T7408
Trifluoroacetic Acid, Optima™ LC/MS Grade, Fisher Chemical™	Fisher Scientific	10266617
Trypsin Sequencing Grade, modified	Merck	11418025001
Water, HiPerSolv CHROMANORM® for LC-MS, suitable for UPLC/UHPLC instruments	VWR	83645.29

**Critical commercial assays**		

BODIPY™ 493/503 (4,4-Difluoro-1,3,5,7,8-Pentamethyl-4-Bora-3a,4a-Diaza-s-Indacene)	Invitrogen™	D3922
BODIPY™ 500/510 C1, C12 (4,4-Difluoro-5-Methyl-4-Bora-3a,4a-Diaza-s-Indacene-3-Dodecanoic Acid)	Invitrogen™	D3823
CD14 MicroBeads, human	Miltenyi	130-050-201
DC Protein Assay II	Bio-Rad	5000112
HCS LipidTOX™ Red Neutral Lipid Stain, for cellular imaging	Invitrogen™	H34476
Human IL-1β DuoSet ELISA	R&D Systems	DY401
Human IL-6 DuoSet ELISA	R&D Systems	DY206
Human IL-8 DuoSet ELISA	R&D Systems	DY208
Human TNFα DuoSet ELISA	R&D Systems	DY210
Micro BCA™ Protein Assay Kit	ThermoFisher	23235
MitoSOX™ Red	ThermoFisher	M36008
MitoTracker™ Green FM	ThermoFisher	M7514
nCounter® Human Metabolic Pathways Panel	Nanostring	XT-CSO-HMP1-12
nCounter® Human Myeloid Innate Immunity Panel	Nanostring	XT-CSO-HMII2-12
pHrodo™ Red E. coli BioParticles™ Conjugate for Phagocytosis	ThermoFisher	P35361
Pierce™ Quantitative Fluorometric Peptide Assay	ThermoFisher	23290

**Deposited data**		

Raw data for NanoString nCounter® Human Metabolic Pathways Panel	NCBI Gene Expression Omnibus (GEO)	GSE255271
Raw data for NanoString nCounter® Human Myeloid Innate Immunity Panel	NCBI Gene Expression Omnibus (GEO)	GSE255273
Raw data for Proteomics analysis	EMBL-EBI Proteomics Identification Database (PRIDE)	PXD050094

**Software and algorithms**		

clusterProfiler 4.2.2	Guangchuang Yu	https://doi.org/10.18129/B9.bioc.clusterProfiler
FlowJo 10.8.0	Tree Star	www.flowjo.com
GraphPad Prism 9	GraphPad Software, Inc.	www.graphpad.com
H. sapiens org.Hs.eg.db, 3.18.0	Marc Carlson	https://doi.org/10.18129/B9.bioc.org.Hs.eg.db
NovoExpress 1.4.1	Agilent	https://www.agilent.com/en/product/research-flow-cytometry/flow-cytometry-software/novocyte-novoexpress-software-1320805
nSolver 4.0	Nanostring	www.nanostring.com/ncounterpro
OpenMS 2.5.0	TOPPAS	https://openms.de/
Perseus 1.6.15.0	MaxQuant	https://maxquant.net/perseus/
RStudio 2023.09.1+494	Posit, PBC	https://posit.co/download/rstudio-desktop/
Seahorse Wave Software 2.6	Agilent	https://www.agilent.com/en/product/cell-analysis/real-time-cell-metabolic-analysis/xf-software/seahorse-wave-desktop-software-740897
Spectronaut 16.2.220903.53000	Biognosys	https://biognosys.com/software/spectronaut/

**Other**		

Ambion™ Nuclease-Free Water (not DEPC-Treated)	ThermoFisher	AM9937
HBSS, no calcium, no magnesium, no phenol red	Gibco	14175053
Phosphate buffered saline (PBS)	Gibco	10010001
RPMI 1640 Medium, GlutaMAX™ Supplement	Gibco	61870036
S-Trap™ mini columns (100 - 300 μg)	Protifi	CO2-mini-80
Seahorse XF base medium	Agilent	102353-100

### Resource availability

#### Lead contact

Further information and requests for resources and reagents should be directed to and will be fulfilled by the lead contact, April Rees (april.rees@swansea.ac.uk).

#### Materials availability

This study did not generate new unique reagents.

#### Data and code availability

NanoString data have been deposited at GEO, and proteomics data at PRIDE, and are publicly available as of the date of publication. Accession numbers are listed in the key resources table. This paper does not report original code. Any additional information required to reanalyze the data reported in this paper is available from the lead contact upon request.

### Experimental model and study participant details

#### Human participants

Peripheral blood was collected from healthy, non-fasted volunteers. These were females aged 18-40, and predominantly Caucasian. Individuals with infection, inflammatory conditions, or obesity (BMI ≥ 30 kg/m^2^) were excluded. Samples were collected with informed written consent and ethical approval was provided by a Health Research Authority (HRA) Research Ethics Committee (13/WA/0190). We did not take stage of menstrual cycle into account, nor the use of hormonal contraception, given the key research question of this study and the strong phenotypic differences between non-pregnant women and women in the latter stages of pregnancy. However, we now have a programme of work exploring effects of menstrual cycle on similar parameters and inflammation more broadly.^66^

#### Patient samples

Peripheral blood was collected from healthy, non-fasted pregnant females at term (37+ weeks), aged 18-40, and predominantly Caucasian. The pregnant females recruited onto the study had a caesarean section within 1-3 days of providing the blood sample, with healthy birth outcomes. Only those undergoing scheduled/elective caesarean section were included to avoid the effect of labour. Exclusion criteria include: multiparous pregnancies, obesity (BMI ≥ 30 kg/m^2^ before pregnancy), adverse obstetric outcomes (e.g., gestational diabetes, hypertension), infection, and inflammatory conditions. Samples were collected with informed written consent and ethical approval was provided by a Health Research Authority (HRA) Research Ethics Committee (11/WA/0040).

#### Cell isolation

Heparinized blood was diluted 1 in 4 with phosphate buffered saline (PBS; Life Technologies) before layering onto Lymphoprep ^TM^ (Stem Cell Technologies, UK) and centrifuged at 400 × *g* for 40 min at room temperature (RT). Peripheral blood mononuclear cells (PBMCs) were collected and washed with RPMI 1640 and Glutamax (Life Technologies, Paisley, UK) twice by centrifugation at 515 × *g* RT. Monocytes were isolated from PBMCs with CD14 magnetic microbeads (Miltenyi) according to the manufacturer’s instructions, using an autoMACS Pro Separator (Miltenyi).

### Method details

#### Flow cytometry

All flow cytometry data was acquired using the ACEA NovoCyte flow cytometer and analyzed using FlowJo^TM^ (version 10.1; BD Biosciences), where compensation was applied to address any spectral overlap. Appropriate controls were used: unstained and single stains to correct for fluorescence spill over. Quality control (QC) particles (Agilent) were used daily to reduce inter-session instrument variability. Details of all antibodies used can be seen in the key resources table.

Monocytes were checked for purity with anti-CD14 (Pacific Blue ^TM^); due to the effect of platelets, particularly on metabolism,^67^ only samples with less than 10% platelets (based on their FSC vs SSC profile) were used for further analysis.

CD16^+^ subsets were identified using anti-CD16 (vioBlue ^TM^) and expression of phenotypic markers assessed using various antibodies.

Phagocytosis was measured using pHrodo^TM^ red *E. coli* BioParticles^TM^ (ThermoFisher) used at either 10 μg/ml, or 25 μg/ml. Cells were incubated for 1 hr at 37°C, 5% CO_2_.

Mitochondrial content of monocytes was monitored using 20 nM MitoTracker^TM^ Green (Life Technologies), incubated for 30 mins at 37°C, 5% CO_2_. For mitochondrial ROS staining, cells were incubated with 5μM MitoSOX^TM^ Red (Life Technologies) for 15 mins at 37°C, 5% CO_2_.

Kynurenine uptake assay was performed as set out by Sinclair et al.^46^ Controls used were unstained, 4°C, an inhibitor of system L amino acid transporters (10 mM BCH; Sigma) and lysine (5 mM; Sigma). Monocytes (5.0 × 10^5^) were first stained with anti-CD16 (FITC) as described previously. After centrifugation cells were resuspended in 200 μl warmed Hanks’ Balanced Salt Solution (HBSS; Life Technologies). All solutions were kept at 37°C. Kynurenine (200 μM; Tocris) was added to the samples with or without the individual controls and uptake was allowed to take place for 4 mins before stopped with 4% paraformaldehyde (PFA; Sigma). Samples were incubated for 30 mins at 37°C, 5% CO_2_ before two washes with FACS buffer.

LipidTOX ^TM^ (Life Technologies) was prepared as per manufacturer’s guidelines. Lipid storage was measured using BODIPY 493 (1 μg/ml; ThermoFisher), incubated for 1 hr at 4°C. Lipid uptake was determined using BODIPY 500 (1 μg/ml; ThermoFisher), incubated for 20 mins at 37°C.

#### Cell lysate preparation and NanoString© nCounter ® analysis

##### Sample processing

1 × 10^6^ of monocytes were washed with 1 ml RPMI 1640/Glutamax and centrifuged at 8,000 × *g* for 4 mins. The cells were re-suspended with diluted RLT buffer (Qiagen, UK) (1/3 with RNase-free water; Invitrogen) to 10,000 cells / μl, and frozen at -80°C.

##### NanoString©

50,000 lysed cells were used for the hybridization reaction performed as per the manufacturer’s instructions (NanoString©), using the nCounter® Myeloid Innate Immunity Panel and the Metabolic Pathways Panel. Hybridized samples were run on the nCounter® SPRINT (NanoString©, USA). Data analysis was performed using the advanced analysis package (2.0.134) within the nSolver© analysis software (Version 4.0, NanoString©), and differential expression p-value adjustment was done with Benjamini-Hochberg. Significant gene changes were determined to be p ≤ 0.05 with a fold change of ≤ -0.5 or ≥ 0.5.

#### Proteomic analysis

##### Sample preparation

1 × 10^6^ of monocytes were washed with 1 ml HBSS and centrifuged at 17,000 × *g* at 4°C for 20 secs twice. The pellet was snap frozen in liquid nitrogen for 10 secs before storage at −80°C.

##### S-trap sample processing

Cell pellet was resuspended in 200 μL SDS lysis buffer (10% SDS, 100 mM triethylammonium bicarbonate [TEAB], pH 7.55) before sonication for 15 min (30 secs on, 30 secs off, 100% amplitude). After sonication, aliquot of the samples was used for protein quantification using the Micro BCA Protein Assay (Thermo Scientific). 300 μg protein was used for further processing. Samples were centrifuged (8 mins, 13,000 × *g*) and the supernatant transferred to fresh tubes. Dithiothreitol (DTT; 20 mM; Thermo Scientific) was added to the supernatant, incubated (10 mins, 95°C), centrifuged (15 secs, 13,000 × g) to collect condensate, and allowed to cool to room temperature. Iodoacetamide (40 mM; Thermo Scientific) was added to the supernatant, incubated (dark, 30 mins, RT), centrifuged (8 mins, 13,000 × *g*), and supernatant moved to a fresh tube. 12% phosphoric acid was added to the supernatant at 1:10, with 350 μl S-Trap Binding buffer (90% aqueous methanol containing a final concentration of 100 mM TEAB, pH 7.1). Samples were processed with S-Trap micro columns (Protifi). Samples were digested overnight at 37°C with 7.5 μg trypsin (Thermo Scientific) in 150 μl digestion buffer (50 mM TEAB), with a further digestion with the same amount of trypsin for 4 hrs the following day. Peptides were extracted and dried under vacuum. The peptides were then resuspended to 50 μl with 1% Formic Acid (Thermo Fisher) and quantified using the Pierce Quantitative Fluorometric Peptide Assay (Thermo Fisher).

##### Data-independent analysis (DIA) mass spectrometry

1.0 μg peptide was analyzed per sample. Samples were injected onto a nanoscale C18 reverse-phase chromatography system (UltiMate 3000 RSLC nano, Thermo Scientific) then electrosprayed into an Q Exactive HF-X Mass Spectrometer (Thermo Scientific). For liquid chromatography buffers were as follows: buffer A (0.1% formic acid in Milli-Q water (v/v)) and buffer B (80% acetonitrile and 0.1% formic acid in Milli-Q water (v/v). Sample were loaded at 10 μl/min onto a trap column (100 μm × 2 cm, PepMap nanoViper C18 column, 5 μm, 100 Å, Thermo Scientific) equilibrated in 0.1% trifluoroacetic acid (TFA). The trap column was washed for 3 min at the same flow rate with 0.1% TFA then switched in-line with a Thermo Scientific, resolving C18 column (75 μm × 50 cm, PepMap RSLC C18 column, 2 μm, 100 Å). The peptides were eluted from the column at a constant flow rate of 300 nl/min with a linear gradient from 3% buffer B to 6% buffer B in 5 min, then from 6% buffer B to 35% buffer B in 115 min, and finally to 80% buffer B within 7 min. The column was then washed with 80% buffer B for 4 min and re-equilibrated in 35% buffer B for 5 min. Two blanks were run between each sample to reduce carry-over. The column was kept at a constant temperature of 40°C.

The data was acquired using an ESI spray source operated in positive mode with spray voltage at 3.200 kV, and the ion transfer tube temperature at 250°C. The MS was operated in DIA mode. A scan cycle comprised a full MS scan (m/z range from 350-1650), with RF lens at 40%, AGC target set to custom, normalized AGC target at 300, maximum injection time mode set to custom, maximum injection time at 20 ms and source fragmentation disabled. MS survey scan was followed by MS/MS DIA scan events using the following parameters: multiplex ions set to false, collision energy mode set to stepped, collision energy type set to normalized, HCD collision energies set to 25.5, 27 and 30, orbitrap resolution 30000, first mass 200, RF lens 40, AGC target set to custom, normalized AGC target 3000, maximum injection time 55 ms.

Data for both MS and MS/MS scans were acquired in profile mode. Mass accuracy was checked before the start of samples analysis.

##### Data analysis

Analysis of the DIA data was carried out using Spectronaut (version 16.2.220903.53000, Biognosys, AG). The direct DIA workflow, using the default settings (BGS Factory Settings) with the following modifications was used: decoy generation set to mutated; Protein LFQ Method was set to QUANT 2.0 (SN Standard) and Precursor Filtering set to Identified (Qvalue); Cross-Run Normalization was unchecked; Precursor Qvalue Cutoff and Protein Qvalue Cutoff (Experimental) set to 0.01; Precursor PEP Cutoff set to 0.1 and Protein Qvalue Cutoff (Run) set to 0.05.

For the Pulsar search the settings were: maximum of 2 missed trypsin cleavages; PSM, Protein and Peptide FDR levels set to 0.01; scanning range set to 300-1800 m/z and Relative Intensity (Minimum) set to 5%; cysteine carbamidomethylation set as fixed modification and acetyl (N-term), deamidation (asparagine, glutamine), dioxidation (methionine, tryptophan), glutamine to pyro-Glu and oxidation of methionine set as variable modifications. The database used was *H. sapiens* downloaded from uniprot.org on 2021-10-11 (77,027 entries).

Further data analysis was performed using Perseus (version 1.6.15.0, https://maxquant.net/perseus/) to generate copy numbers and in Microsoft Excel Office 365 to generate protein fold-change values. Protein values were exported onto R (Version 2022.07.1, RStudio) to create z-score values and for further analysis.

Significant protein changes were determined to be p ≤ 0.05 with a fold change of ≤ −0.5 or ≥ 0.5.

For the Gene Set Enrichment Analysis (GSEA), the clusterProfiler package (version 4.2.2)^68^^,^^69^ on R was used with the gseGO function and the database for *H. sapiens* (org.Hs.eg.db downloaded from Bioconductor.org). Pathways analyzed for biological processes and molecular functions had a gene size minimum of 25, a maximum of 250, and a p value cut off 0.05. The mass spectrometry proteomics data have been deposited to the ProteomeXchange Consortium via the PRoteomics Identification DatabasE (PRIDE) partner repository with the dataset identifier PXD050094.

#### Bioenergetic analysis

Bioenergetic analysis was carried out using the Seahorse Extracellular Flux Analyzer XFe96 (Agilent Technologies), optimized as in Jones et al.^10^ Monocytes (2.0 × 10^5^ cells/well) in XF assay media (minimal Dulbecco’s modified eagle medium [DMEM]; Agilent) supplemented with 5.5 mM glucose (Agilent), 1 mM pyruvate (Agilent) and 2 mM glutamine (Sigma) were seeded onto a Cell-Tak (22.4 μg/ml; Corning) coated microplate.^70^ Parameters for oxidative phosphorylation (OXPHOS) and glycolysis were measured simultaneously via oxygen consumption rate (OCR; pmoles/min) and extracellular acidification rate (ECAR; mpH/min) respectively with use of injections: oligomycin (1 μM), FCCP (1 μM), antimycin A and rotenone (both 1 μM) and monensin (20 μM; activation assay only) (all from Sigma).

#### Cardiolipin quantification

1 × 10^6^ monocytes were aliquoted and washed with 1 ml RPMI 1640/Glutamax, centrifuged at 400 × *g* for 5 mins. The pellet was then washed twice with PBS and centrifuged using the same conditions. PBS was removed completely, and the pellet was frozen at -80°C until use. Upon thawing, cells were washed with 100 μl of dH2O and centrifuged at 400 × *g* for 5 mins, with the supernatant being completely removed. The pellet was carefully re-suspended in 50 μl of dH2O. Protein concentration was calculated using the detergent compatible (DC) protein assay (Bio-Rad). 10 μg of protein was used for lipid extraction, performed using the miniextraction method.^71^ Samples were spiked with a tetramyristoyl cardiolipin (CL 14:0) as internal standard (Avanti Polar Lipids) at 1.25 nmoles/μg of protein. Extracted lipids were mixed in a 1:1 ratio with 30 mg/ml of 9-aminoacridine (9-AA; Acros Organics) matrix solution. 9-AA was reconstituted with 2-propanol-acetonitrile (60:40; Fisher Scientific). 1 μl of the lipid:matrix solution was spotted onto the target plate (MTP 384 target plate ground steel BC; Bruker). An UltrafleXtreme MALDI-ToF mass spectrometer (Bruker, Germany) was used to acquire the spectra, operating in the negative polarity. Acquisition included a variable number of shots between 4000-5000 with accumulation stopped when the peak of the internal standard reached roughly 5K AU.

Data was exported to OpenMS© software (Version 2.5.0, TOPPAS) where peaks were identified as specific lipids using their m/z value with the assistance of LIPID MAPS® (https://www.lipidmaps.org/) and in agreement with previous literature.^71^ The intensity of the peaks was used for analysis. Quantification was done by comparing peak intensities of the endogenous cardiolipin species with that of the internal standard with known quantity, including isotope correction.

#### Cell culture

Monocytes (2.0 × 10^5^ cells/well) in RPMI 1640, 10% autologous plasma and 2-mercaptoethanol (2-ME) were left unstimulated or stimulated with LPS (10 ng/ml; Invivogen), or muramyl-dipeptide (MDP; 1 μg/ml; Invivogen) at 37°C in 5% CO_2_-in-air for 24 h. Autologous plasma was used instead of FBS to provide a semblance of the native non-pregnant or pregnant environment, to more closely represent the function of the cells *in vivo*. Supernatants were harvested for cytokine analysis, and cells used directly for bioenergetic analysis.

#### Cytokine analysis

ELISAs were carried out as per the manufacturer’s instructions: IL-1β, TNFα, IL-6, IL-8, and IL-10 (DuoSets; R&D Systems, Bio-Techne). Due to the use of autologous plasma, cytokine measurements observed in media only were checked, but no detectable cytokines were present.

### Quantification and statistical analysis

Statistical analysis was performed using GraphPad Prism© V9 (Dotmatics). Data are represented as the mean +/- standard error of the mean (SEM). To test for normality, the one-sample Kolmogorov-Smirnov (K-S) test. A Mann-Whitney test was used if the data was non-parametric for comparison between two sets of data, or a two-way ANOVA for further groups with a Šidák’s multiple comparisons post-hoc test. All experiments have replicate sample size at minimum of n = 3, and a p ≤ 0.05 was determined to be significant. Specific details can be found in the figure legends.
