# Supplementary material for: Immunometabolic adaptation in monocytes underpins functional changes during pregnancy
Source: iScience. 2024 Apr 18;27(5):109779. doi: 10.1016/j.isci.2024.109779 (PMC11088341; doi:10.1016/j.isci.2024.109779)
Supplement: Document S1. Figures S1–S4 and Tables S1–S3 [file mmc1.pdf]

iScience, Volume *27*

Supplemental information

Immunometabolic adaptation in monocytes underpins functional changes during pregnancy

April Rees, Benjamin J. Jenkins, Roberto Angelini, Luke C. Davies, James G. Cronin, Nicholas Jones, and Catherine A. Thornton

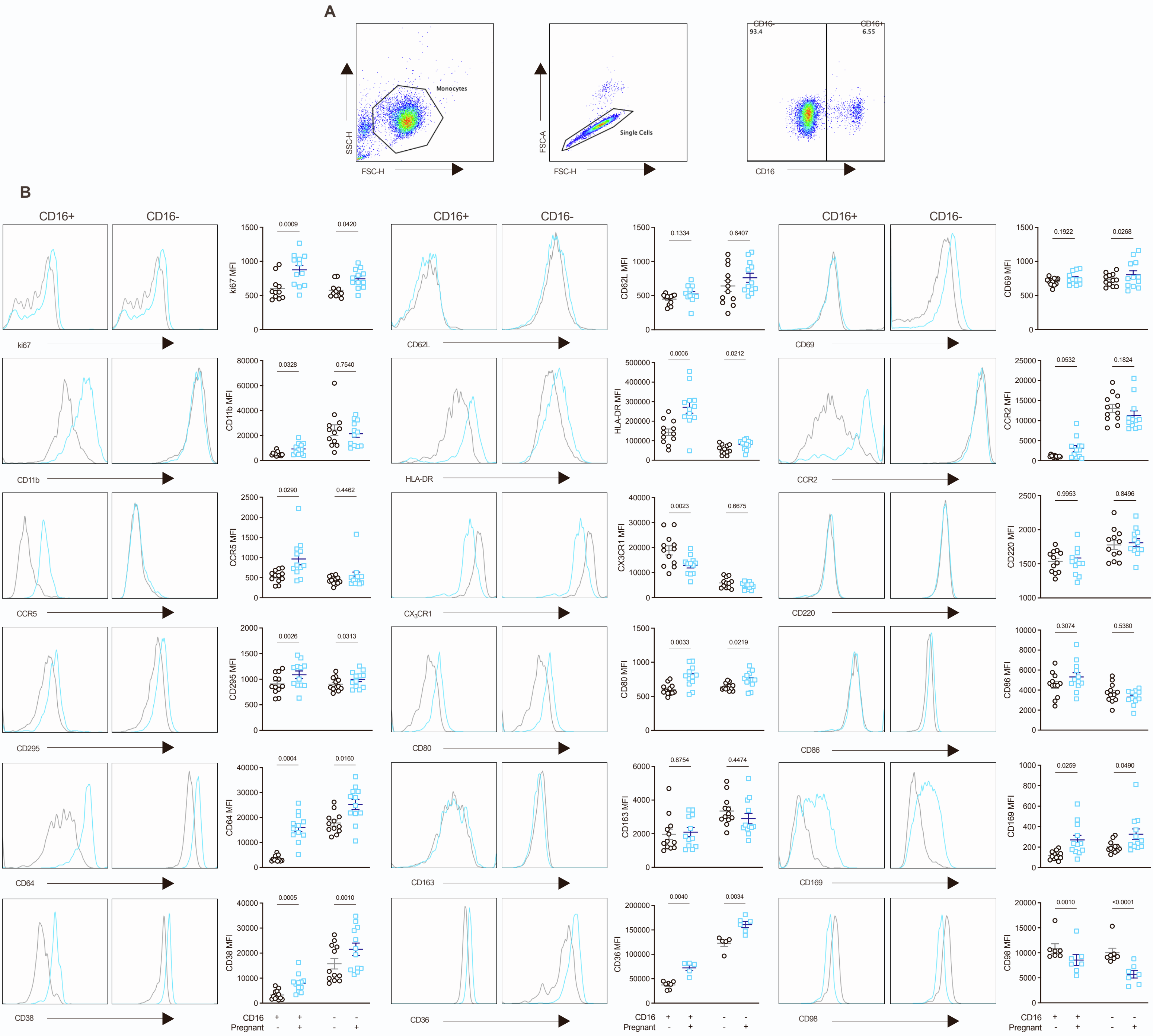

Figure S1. Relating to Figure 1, representative dot plots and histograms for flow cytometry phenotyping. (A) Dot plots illustrating the gating strategy for isolated monocytes to determine the phenotype of CD16+ and CD16- subsets. (B) Histograms and individual graphs for each expression marker analysed for CD16+ and CD16- subsets from non-pregnant (grey) and pregnant (blue) monocytes.

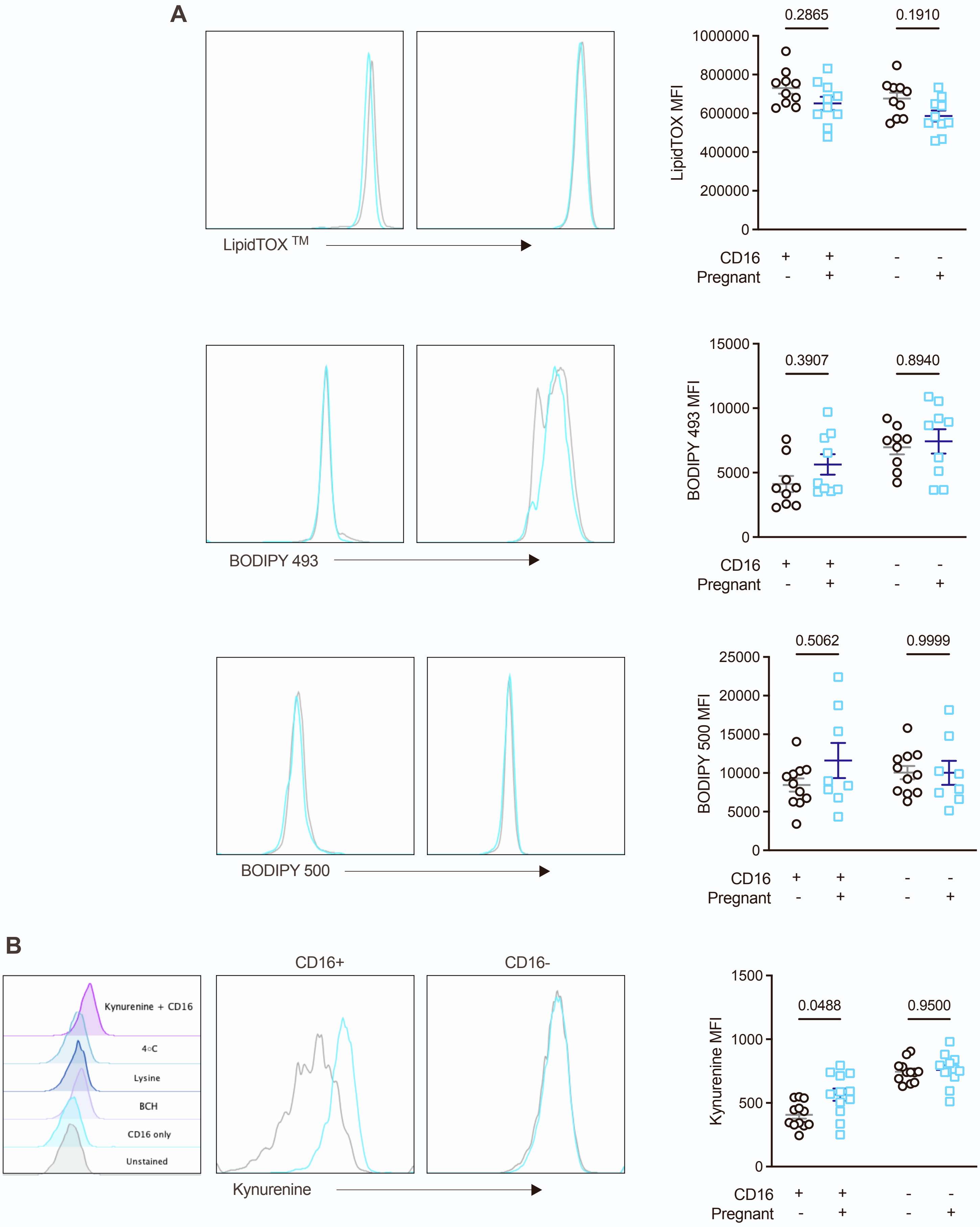

Figure S2. Relating to Figure 1, Functional consequences of the changes in CD36 and CD98. (A) Monocytes from non-pregnant (grey) and pregnant (blue) donors were subjected to LipidTOX TM, BODIPY 493 (both storage), and BODIPY 500 (uptake), and analysed on the flow cytometer. (B) Representative histograms for the appropriate controls for analysis of kynurenine uptake. Monocytes were analysed for their ability to take kynurenine up from non-pregnant (grey) and pregnant (blue) donors.

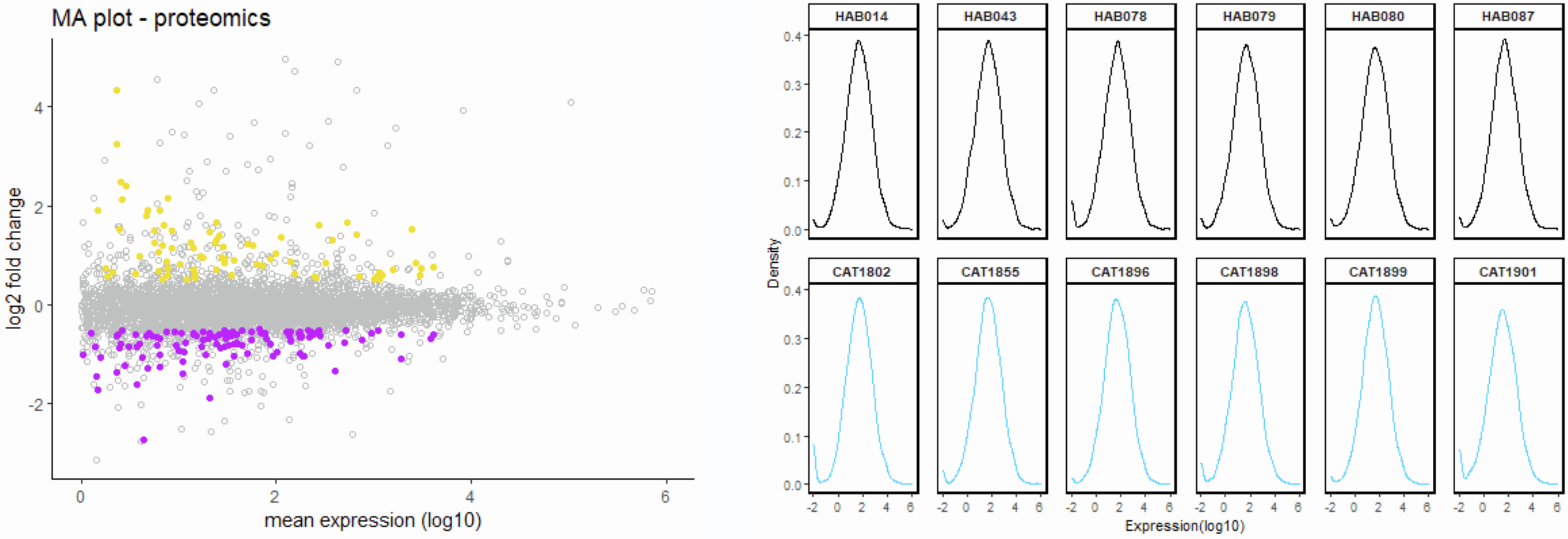

Figure S3. Relating to Figure 1, QC checks for proteomics. An MA plot for the proteomics data showing proteins which were found to be significantly up-regulated in pregnancy in gold, and down-regulated in purple. Distribution plots of the proteins found for each donor are also illustrated, highlighting the consistency between the groups.

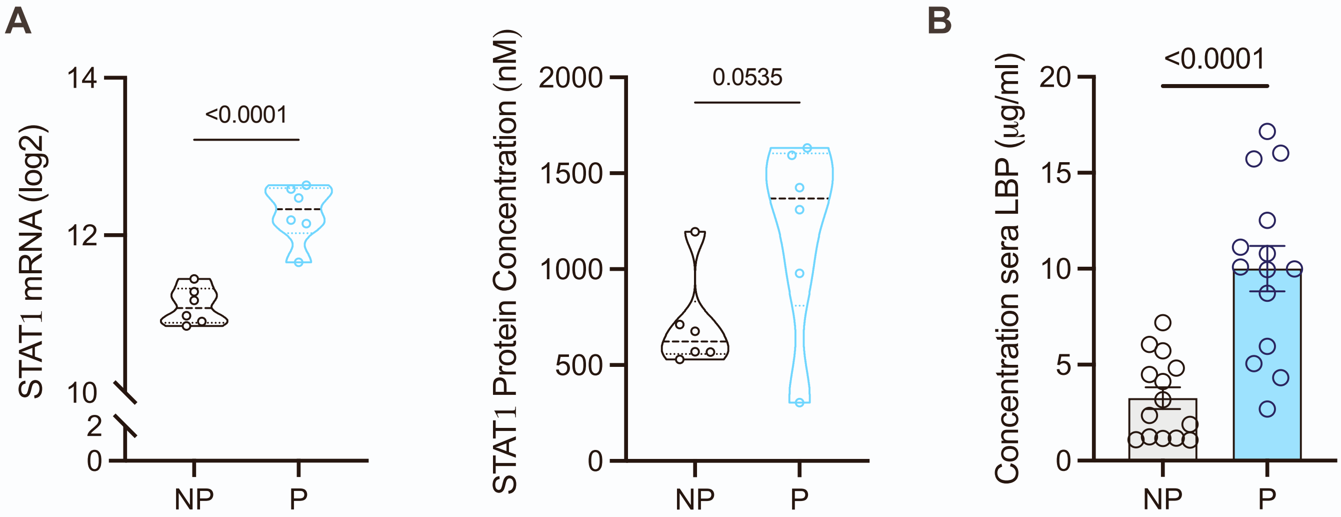

Figure S4. Relating to Figure 1 and Figure 2, STAT1 mRNA and protein, and LBP levels in serum from non-pregnant and pregnant females. (A) STAT1 mRNA data taken from the NanoString (C) Metabolism panel, and protein data taken from proteomics. (B) LBP levels in serum (n=14/group) were measured using an LBP-specific ELISA. Statistical analysis was performed using a Mann-Whitney test where p < 0·05 was considered significant.

Table S1, Relating to Figure 1. Significantly up-regulated genes from the NanoString © Myeloid Innate Immunity panel.

Gene ID	Gene description	Log2 fold change	p value
PSME2	Proteasome activator complex subunit 2	1.190	0.0070
STAT1	Signal transducer and activator of transcription 1	1.620	0.0070
TNFSF10	TNF Superfamily Member 10	1.600	0.0070
IRF7	Interferon regulatory factor 7	1.160	0.0080
FCGR3A	Fc Gamma Receptor IIIa	1.270	0.0080
VRK2	Serine/threonine-protein kinase	0.853	0.0094
ISG15	Ubiquitin Like Modifier	1.650	0.0100
FBP1	Fructose-Bisphosphatase 1	0.695	0.0106
PSMB9	Proteasome subunit beta type-9	0.836	0.0106
CBR1	Carbonyl Reductase 1	0.534	0.0106
TAP1	Antigen Peptide Transporter 1	1.240	0.0106
TRAFD1	TRAF-type zinc finger domain containing 1	0.727	0.0108
TGM2	Transglutaminase 2	2.340	0.0108
ARMC1	Armadillo Repeat Containing 1	0.647	0.0108
C3AR1	Complement C3a Receptor 1	0.933	0.0108
SIGLEC1	Sialic Acid Binding Ig Like Lectin 1	2.730	0.0115
IRF2	Interferon Regulatory Factor 2	0.758	0.0120
GPR65	G Protein-Coupled Receptor 65	0.899	0.0120
CCR2	C-C Motif Chemokine Receptor 2 (MCP-1 receptor)	1.700	0.0133
CD38	Cyclic ADP ribose hydrolase	2.210	0.0138
FPR2	Formyl Peptide Receptor 2	1.790	0.0150
TLR10	Toll-like receptor 10	1.320	0.0200
CTSL	Cathepsin L	1.520	0.0221
FCGR1A	Fc Gamma Receptor Ia	1.900	0.0221
IFIT1	Interferon-induced protein with tetratricopeptide repeats 1	2.560	0.0235
TLR1	Toll-like receptor 1	1.070	0.0254
FADD	Fas Associated Via Death Domain	0.910	0.0259
C1QB	Complement C1q B Chain	2.310	0.0259
RNASE3	Ribonuclease 3	0.619	0.0259
USP18	Ubiquitin Specific Peptidase 18	1.920	0.0260
IRF1	Interferon regulatory factor 1	0.857	0.0277
CASP10	Cysteine-aspartic acid protease 10	0.753	0.0281
PSMB8	Proteasome subunit beta type-8	0.529	0.0296
P2RX1	Purinergic Receptor P2X 1	0.808	0.0325
C1QC	Complement C1q C Chain	2.630	0.0327
TLR7	Toll-like receptor 7	1.130	0.0333
TNFRSF1A	Tumor necrosis factor receptor superfamily member 1A	0.585	0.0333
IFNAR2	Interferon Alpha and Beta Receptor Subunit 2	0.519	0.0341
MX2	MX Dynamin Like GTPase 2	0.962	0.0392
CXCR2	C-X-C Motif Chemokine Receptor 2 (IL-8 receptor)	0.982	0.0404
CASP5	Cysteine-aspartic acid protease 5	1.010	0.0441
MX1	MX Dynamin Like GTPase 1	1.620	0.0484

Table S2, Relating to Figure 1. Significantly down-regulated genes from the NanoString © Myeloid Innate Immunity panel.

Gene ID	Gene description	Log2 fold change	p value
CD83	Member of Ig superfamily	-1.850	0.0069
NFKBIZ	NF-kappa-B inhibitor zeta	-1.400	0.0080
OSM	Oncostatin M	-1.850	0.0080
IL1A	Interleukin-1 alpha	-5.420	0.0080
ATF3	Activating transcription factor 3	-2.150	0.0096
PTGS2	Prostaglandin-endoperoxide synthase 2 (COX-2)	-1.220	0.0096
GPR183	G Protein-Coupled Receptor 183	-2.500	0.0106
TNF	Tumour necrosis factor	-1.790	0.0106
ADORA2A	Adenosine A2A receptor	-1.230	0.0106
CCL20	Macrophage Inflammatory Protein-3	-4.930	0.0106
FOSL1	Fos-related antigen 1	-2.440	0.0106
ICAM1	Intercellular Adhesion Molecule 1	-0.937	0.0111
IL1B	Interleukin-1 beta	-3.610	0.0114
NFKBIA	NF-kappa-B inhibitor alpha	-1.860	0.0120
VEGFA	Vascular endothelial growth factor A	-1.350	0.0127
IER3	Immediate Early Response 3	-1.730	0.0133
PTX3	Pentraxin-related protein	-2.070	0.0133
PLAUR	Plasminogen activator, urokinase receptor	-1.120	0.0221
MAFF	MAF Basic Leucine Zipper Transcription Factor F	-3.000	0.0221
CCRL2	C-C chemokine receptor-like 2	-0.914	0.0221
NAMPT	Nicotinamide phosphoribosyltransferase	-1.150	0.0221
NLRP3	NLR Family Pyrin Domain Containing 3	-1.000	0.0235
CXCL8	Interleukin 8	-1.590	0.0239
TNFAIP3	Tumor necrosis factor, alpha-induced protein 3	-1.730	0.0259
NR4A2	Nuclear receptor 4A2	-2.450	0.0259
CD99	Single-chain type-1 glycoprotein	-0.775	0.0259
FEM1C	Fem-1 Homolog C	-0.643	0.0259
NFKBIE	NF-kappa-B inhibitor epsilon	-0.834	0.0260
C5AR1	Complement C5a Receptor 1	-0.627	0.0312
DUSP2	Dual Specificity Phosphatase 2	-2.090	0.0325
CXCL2	C-X-C Motif Chemokine Ligand 2	-1.820	0.0333
CYTIP	Cytohesin 1 Interacting Protein	-0.647	0.0342
EMP1	Epithelial Membrane Protein 1	-1.470	0.0359
LIF	Leukemia inhibitory factor/IL-6 class cytokine	-4.710	0.0367
BCL6	Transcription Repressor	-0.670	0.0392
CXCL3	C-X-C Motif Chemokine Ligand 3	-2.030	0.0441
BCL2	Outer mitochondrial membrane apoptosis regulator	-1.080	0.0457
CDKN1A	Cyclin-dependent kinase inhibitor 1A	-1.270	0.0471
RIPK2	Receptor-interacting protein of serine/threonine protein kinases	-1.130	0.0483
CCL4	C-C Motif Chemokine Ligand 4	-3.380	0.0483

Table S3, Relating to Figure 2. Significantly up- and down-regulated genes from the NanoString © Metabolic Pathways panel.

	Gene ID	Gene description	Log2 fold change	p value
**Upregulated**	IDH1	Isocitrate dehydrogenase 1	0.614	0.0005
	TLR7	Toll-like receptor 7	1.150	0.0020
	STAT1	Signal transducer and activator of transcription 1	1.200	0.0034
	IRF1	Interferon regulatory factor 1	0.831	0.0044
	PSME2	Proteasome activator complex subunit 2	0.846	0.0051
	ACSF3	Acyl-CoA synthetase family member 3	0.537	0.0051
	RIMKLB	Ribosomal Modification Protein RimK Like Family Member B	0.962	0.0108
	JAK2	Janus kinase 2	0.596	0.0108
	PDCD1LG2	Programmed cell death 1 ligand 2	2.080	0.0118
	RBBP5	Retinoblastoma-binding protein 5	0.503	0.0125
	MYC	Myc	0.830	0.0144
	KMO	Kynurenine 3-monooxygenase	0.611	0.0144
	MSRB2	Methionine Sulfoxide Reductase B2	0.567	0.0184
	SLC6A12	Sodium- and chloride-dependent betaine transporter	0.842	0.0184
	PNOC	Prepronociceptin	1.490	0.0188
	SCD	Stearoyl-CoA desaturase	1.370	0.0233
	SOS1	Ras and Rac guanine nucleotide exchange factor	0.509	0.0321
	TYMP	Thymidine phosphorylase	0.682	0.0458
	MS4A4A	Membrane Spanning 4-Domains A4A	0.857	0.0492
**Downregulated**	FANCI	FA Complementation Group I	-0.574	0.0184
	VEGFA	Vascular endothelial growth factor A	-1.080	0.0196
	APOM	Apolipoprotein M	-0.524	0.0470
	PRR5	Proline Rich 5	-0.937	0.0492
	ADORA2A	Adenosine A2A receptor	-0.870	0.0515
